# Pre‐folding purification procedures for inclusion body‐derived non‐tagged cationic recombinant proteins with multiple disulfide bonds for efficient refolding

**DOI:** 10.1002/btpr.3532

**Published:** 2025-01-25

**Authors:** Shuichiro Kimura, Wataru Yamamoto, Ai Miyamoto, Koreyoshi Imamura, Junichiro Futami

**Affiliations:** ^1^ Division of Applied Chemistry, Graduate School of Natural Science and Technology Okayama University Okayama Japan; ^2^ Graduate School of Interdisciplinary Science and Engineering in Health Systems Okayama University Okayama Japan

**Keywords:** chemical modification, protein refolding, reversed‐phase HPLC, VEGF

## Abstract

The production of disulfide‐containing recombinant proteins often requires refolding of inclusion bodies before purification. A pre‐refolding purification step is crucial for effective refolding because impurities in the inclusion bodies interfere with refolding and subsequent purification. This study presents a new pre‐refolding procedure using a reversible *S*‐cationization technique for protein solubilization and purification by reversed‐phase high performance liquid chromatography. This pre‐folding purification step improves refolding yield by effectively removing the refolding inhibitors from contaminates from bacterial inclusion bodies, and reducing proteolytically degraded products. Because this procedure does not require a peptide tag for affinity purification, it is a superior technique to subsequently perform a simplified downstream process wherein the affinity tag needs to be removed. This study reports improved refolding and purification procedure to obtain the highly cationic (pI = 9.25) mouse vascular endothelial cell growth factor (188 amino acids form) that is used as a model protein in our study; this protein shows a homodimeric conformation and possesses multiple disulfides.

## 1 INTRODUCTION

Various host cells, ranging from prokaryotic to eukaryotic and mammalian cells, and high‐yield cell‐free protein expression systems have been developed to produce recombinant proteins.^1^, ^2^, ^3^, ^4^ Selection of the most appropriate protein expression system for each protein of interest is a key factor in the production of biologically active recombinant proteins. *Escherichia coli* (*E. coli*) is the most widely used host cell in research and industrial applications owing to its cost‐effectiveness and ease of handling. To date, more than 25% of all recombinant biopharmaceuticals are produced in *E. coli*.^5^ However, obtaining biologically active proteins from *E. coli* is often challenging because of frequent aggregation of recombinant proteins caused by the absence of eukaryotic folding machinery.^6^

Improving the foldability of mammalian proteins in *E. coli* is often achieved by lowering their expression temperature, thereby adapting the faster translational elongation rate of prokaryotes to the slower translational elongation rate of mammalian cells.^6^, ^7^ Another viable strategy to improve protein folding is the coexpression of chaperone proteins.^6^ Regarding purification of disulfide‐containing proteins, the formation of correct disulfide bonds is also required to achieve their biologically active conformation.^6^ To facilitate protein folding in engineered *E. coli* cells, oxidative cytosolic conditions can be created by mutating glutathione reductase and thioredoxin reductases.^8^ Alternatively, the use of bacterial signal peptides for periplasmic secretion has been attempted,^9^ although difficulties are often encountered when such peptides are used, particularly with proteins containing multiple disulfide bonds.

In *E. coli*, misfolded proteins typically aggregate into inclusion bodies (IBs). IBs are advantageous to be utilized for overexpression because they usually resist proteolytic degradation by endogenous bacteria. After cell lysis and thorough washing of IBs, recombinant proteins can be recovered as insoluble fractions by centrifugation or microfiltration.^10^, ^11^ Although the purity of recovered IBs commonly depends on the protein expression level, host cell, and recovery process, IBs from overexpressed proteins can detected as the main band on SDS‐PAGE. These IBs solubilize using high urea or guanidium hydrochloride concentrations as denaturants in general. Alternative solubilizing reagents, such as n‐propanol mixed urea,^12^ SDS,^13^ or N‐lauroylsarcosine,^14^ are also proposed for designing with improved refolding procedures.

Contamination with nucleic acids due to electrostatic interactions must be considered when dealing with cationic proteins because nucleic acids can interfere with refolding or purification processes.^15^, ^16^ Therefore, purification of IB‐derived proteins before performing in vitro refolding experiments is a factor that improves the yield of refolded proteins. Immobilized metal affinity chromatography (IMAC) using a histidine tag (HisTag) is a common choice for this purification step because it allows handling at higher denaturant concentrations. However, removal of the HisTag by a specific protease often diminishes the recovery yield because of non‐specific cleavage by a defined protease. To avoid this issue, it is viable to utilize a reversible Cys residue‐specific *S*‐cationization chemical modification reagent of [3‐(trimethylammonium)propyl] methanethiosulphonate (TAPS‐Sulfonate) is as an alternative strategy^16^, ^17^, ^18^ (Figure S1). The resultant of reversible *S*‐cationized protein produced by TAPS‐Sulfonate exhibits high water solubility. Thus, most bacterial cell‐derived impurities in IBs can be sedimented during the dialysis step.^16^ The resulting reversible *S*‐cationized protein products allow efficient in vitro refolding, in‐cell folding, and intracellular delivery techniques.^19^, ^20^, ^21^

Despite improved refolding procedures for bacterial IBs using reversible *S*‐cationization techniques, two challenges remain. The first challenge refers to tightly bound bacteria‐derived impurities that cannot be completely removed by solubility‐based fractionation. The second is proteolytic degradation of recombinant proteins in IBs. Although commonly used *E. coli* BL21 host strains are selected for their Lon proteinase deficiency^22^, ^23^ certain proteins within IBs undergo proteolytic degradation during expression.

In this study, we present an alternative method for the purification of non‐tagged recombinant proteins with disulfide bonds using reversed‐phase high‐performance liquid chromatography (HPLC), followed by refolding and chromatographic purification of these proteins. The model protein used in this study, mouse vascular endothelial cell growth factor (VEGF), is an essential potent angiogenic growth factor.^24^ VEGF‐A's biophysical properties are highly cationic (pI = 9.25), with three intramolecular disulfide bridges and the formation of homodimers through two additional disulfide bridges conserved in biologically active conformation.^25^, ^26^ Based on the findings obtained in this study, we discuss the feasibility of a TAPS‐Sulfonate‐based approach for the purification of multiple disulfide‐containing proteins.

## 2 MATERIALS AND METHODS

### 2.1 Upstream production methods

#### 2.1.1 Construction of plasmid DNA for recombinant protein expression

A cDNA fragment encoding the mature form of mouse VEGF‐A (uniport_Q00731) comprising 188 amino acids of isoform (Ala27‐Arg214)^27^, ^28^ was synthesized using Integrated DNA Technologies (Coralville, IO, USA). Bacterial recombinant protein expression vectors were designed to produce a non‐tagged mature form or carboxy‐terminal HisTag‐fused form of the pET28b vector (Novagen, Madison, WI, USA). The extracellular domain of mouse VEGF receptor 2 gene (VEGFR2, Uniport _P35918) was amplified by PCR from mouse placental cDNA (GenoStaff, Tokyo, Japan). Mammalian expression vectors were designed to express human IgG1 Fc‐HisTag‐fused protein (VEGFR2‐Fc),^29^ the secreted form of VEGF‐A‐HisTag, and erythropoietin (EPO) under the control of a CMV promoter with an improved gene expression vector.^30^, ^31^

#### 2.1.2 VEGF‐A expression, cell lysis, and production of IBs

*E. coli* BL21 (DE3) (Nippongene, Tokyo, Japan) or Origami 2 (DE3) (Novagen, Madison, WI, USA) were transformed with their respective expression plasmid DNA and cultured on LB agar plates containing 30 μg/mL kanamycin. The bacterial colonies thus obtained were inoculated into 50 mL of LB containing 30 μg/mL kanamycin. After shaking for 4 h at 37°C, the precultures were transferred to two sets of 400 mL Terrific Broth. When cell density reached OD_600_ = 0.8, 0.4 mM isopropyl‐β‐D‐1‐thiogalactopyranoside (IPTG) was added, followed by further incubation for 3 h. Bacterial cells were then harvested by centrifugation, and the pelleted cells were stored at −80°C.

The bacterial cells were resuspended in 80 mL of 50 mM Tris–HCl buffer (pH 7.5) containing 50 mM NaCl and 5 mM MgSO_4_, supplemented with a protease inhibitor cocktail (#03969, Nacalai Tesque, Kyoto, Japan) and disrupted by sonication (Sonifier 250A, Branson) on ice for 3 min at three times repeated. For nucleic acid digestion, 1 μL Benozonase Nuclease HC (#71205, Millipore) was added and incubated for 30 min at 25°C. After recovering the insoluble fraction by centrifugation at 8000 × g for 10 min, the pellets were resuspended gently in 0.15 M NaCl solution by assisting with a sonicator, and the IBs were recovered by centrifugation. For the VEGF‐A‐HisTag protein expressed in Origami2(DE3), the soluble fraction was subjected to immobilized metal affinity chromatography (IMAC) purification after the first centrifugation step.

### 2.2 Process of purification of recombinant proteins

#### 2.2.1 Solubilization of recombinant proteins by reversible *S*‐cationization

Recombinant VEGF‐A protein in IBs was solubilized in 6 M guanidine hydrochloride (GdnHCl) containing 0.1 M Tris–HCl, pH 8.0. After degassing the solution in a round‐bottomed flask, the Cys residues were reduced with 30 mM dithiothreitol (DTT) by incubation for 90 min at 37°C under a nitrogen gas atmosphere. To prepare *S*‐cationized protein by alkyl disulfide conjugation, 90 mM TAPS‐Sulfonate (Katayama Chemical, Osaka, Japan) was added to the mixture, and the mixture was incubated for 30 min at 25°C.^16^, ^32^ After addition of 0.1 volume of acetic acid and 0.1% polyethyleneimine (PEI; average molecular weight of 600, Fujifilm‐Wako, adjusted to pH 7.5 with HCl), the mixtures were extensively dialyzed with pure water for two days at 4°C. The resulting precipitate containing the bacterial protein and nucleic acid complex with PEI was removed by centrifugation, whereas TAPS‐VEGF‐A was recovered in the aqueous phase because of its high net cationic charge.

#### 2.2.2 Purification of VEGF‐A before refolding

Two milligrams of non‐tagged TAPS‐VEGF‐A were purified on a reversed‐phase HPLC column (COSMOSIL Protein‐R, 4.6 mm I.D. × 150 mm, Nacalai Tesque) using an acetonitrile linear gradient (1%–80% for 60 min) elution procedure in the presence of 0.1% HCl, at 0.5 mL/min of flow rate. The loading capacity of *S*‐cationized protein on this column has been observed in ranges of 1–2 mg/injection.^33^, ^34^ This purification procedure was repeated to obtain sufficient amounts of the sample, as the chromatographic separation process was highly reproducible.^33^ The main peaks on HPLC were collected and used for refolding experiments. IBs of VEGF‐A‐HisTag protein expressed in BL21(DE3) cells were purified using IMAC in the presence of a denaturant. Briefly, IBs solubilized in 6 M GdnHCl containing 0.5 M NaCl, 10 mM imidazole, 50 mM phosphate buffer pH 8.0, were loaded onto an Ni‐NTA‐Sepharose column (Qiagen, Hilden, Germany), washed with 40 mM imidazole, and fractions were eluted with 300 mM imidazole in the presence of 8 M urea, 0.3 M NaCl, 50 mM phosphate buffer pH 7.4.

#### 2.2.3 Refolding of VEGF‐A

Denatured forms of purified VEGF‐A protein were refolded using a rapid dilution procedure.^15^ Non‐tagged TAPS‐VEGF‐A or denatured VEGF‐A‐HisTag was directly diluted in refolding buffer consisting of 20 mM Tris–HCl pH 8.0, 2 M urea, 0.1 M NaCl, 30% glycerol, 2 mM reduced glutathione (GSH), and 0.5 mM oxidized glutathione (GSSG) to a final protein concentration of 0.1 mg/mL. Refolding was initiated by rapid dilution with stirring to become the above component of the mixtures at 25°C and continued to incubate for 3 days at 4°C.

#### 2.2.4 Purification of refolded VEGF‐A

The refolded proteins were purified using cation‐exchange column chromatography. After removing misfolded aggregates by centrifugation, the refolded samples were applied to a Resource‐S column (Cytiva, Tokyo, Japan) equilibrated with 20 mM Tris–HCl buffer pH 7.2, at the flow rate of 0.5 mL/min. A linear gradient (0–1 M for 60 min) of NaCl was used to elute the adsorbed proteins, and peak fractions were collected using the AKTA GO chromatography system (Cytiva). The formation of a disulfide‐linked homodimer of VEGF‐A was confirmed on SDS‐PAGE by comparing migration under reducing or non‐reducing conditions. All SDS‐PAGE analyses employed SuperSep Ace, 5%–20% gradient polyacrylamide gel (Fujifilm Wako, Osaka, Japan) with XL‐Ladder Broad marker (Aproscience, Tokushima, Japan) or magic marker XP (Thermo Fisher Scientific, Waltham, MA, USA).

### 2.3 Comparative study to mammalian expression system

#### 2.3.1 Production of secretory recombinant proteins in mammalian cells

Recombinant proteins were expressed in Expi293 cells (Thermo Fisher Scientific) by transient transfection with plasmid DNA and purified by IMAC. Detailed procedures have been described previously.^29^ Because the expressed secretory VEGF‐A‐HisTag protein failed to undergo IMAC purification at the absorption step, it was purified using a HiTrap Heparin column (Cytiva). Contaminated DNAs were detected by PCR using a pair of primers upstream and downstream of a DNA fragment encoding VEGF‐A HisTag or EPO‐HisTag.

### 2.4 Analytical methods

#### 2.4.1 Biological activity of VEGF‐A protein

Pooled human umbilical vein endothelial cells (HUVECs) isolated in Growth Medium 2 were purchased from Takara Bio (Shiga, Japan) and cultured according to the manufacturer's instructions. VEGF‐A–induced HUVEC proliferation was measured after 4 days of incubation using the Cell Counting Kit‐8 (Dojin, Kumamoto, Japan) to evaluate the biological activity of the purified recombinant protein. Cell proliferation rate was determined as cell growth in fully supplemented growth factor mixtures to achieve 100% growth, and cell growth rate was evaluated by adding recombinant VEGF‐A to the basal medium. Commercially available mouse VEGF‐A protein (Fujifilm Wako) was used as a positive control.

For VEGF‐A–induced phosphorylation of VEGFR2 in the HUVEC assay, the cells were starved in the absence of growth supplements for 4 h and stimulated with 25 ng/mL VEGF‐A for 5 min. After washing the cells twice with ice‐cold PBS, cell lysates were prepared in RIPA buffer supplemented with a phosphatase inhibitor cocktail (Nacalai Tesque). Phosphorylation of VEGFR2 was confirmed by western blotting using rabbit phospho‐VEGFR2 (#2478) and VEGFR2 (#2479) monoclonal antibodies (Cell Signaling Technology, Danvers, MA, USA).

## 3 RESULTS AND DISCUSSION

### 3.1 Overexpression of non‐tagged VEGF‐A and solubilization of IBs

**FIGURE 1:**
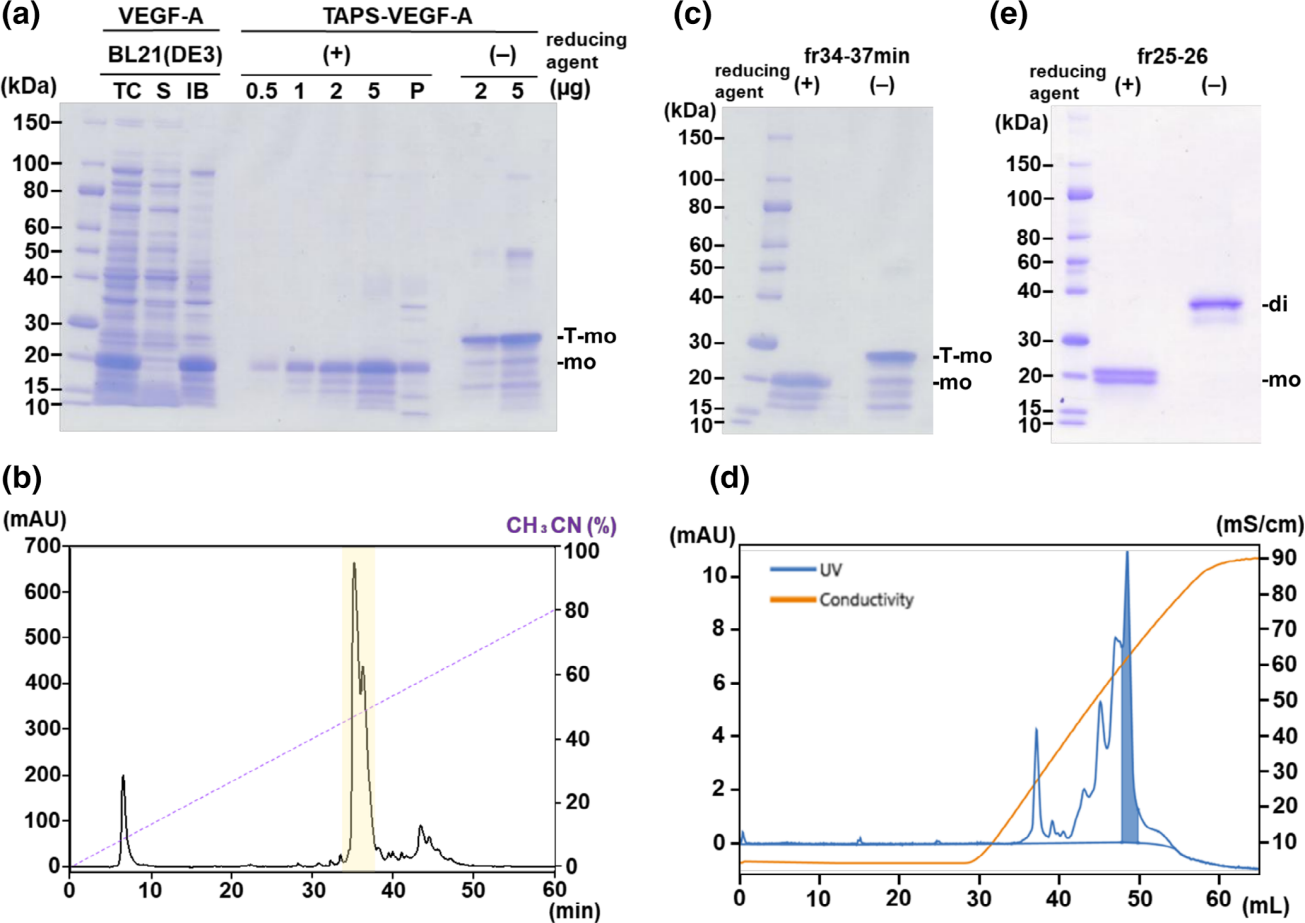
(a) Analysis using SDS‐PAGE of VEGF‐A expressed in *E. coli* and its solubilization by TAPS‐Sulfonate. Total cellular protein (TC) lysate was separated into soluble (S) and insoluble (IB) fractions. VEGF‐A proteins derived from IBs were solubilized by conjugating with TAPS‐Sulfonate and analyzed under reducing (+) and non‐reducing (−) conditions. Bands for monomeric reversibly S‐cationized TAPS‐VEGF‐A (T‐mo) and monomeric VEGF‐A (mo) were indicated on the right side. (b) Purification of pre‐folded TAPS‐VEGF‐A by reversed‐phase HPLC. (c) Fractions of the peak marked in yellow in (b) were collected as refolded material and analyzed by SDS‐PAGE. (d) Purification of refolded VEGF‐A by cation exchange column chromatography. The fraction of the peak marked in blue was collected and analyzed by SDS‐PAGE (e), and the refolding recovery rate was calculated. The band for a homodimeric form of VEGF‐A (di) was indicated on the right side.

Recombinant VEGF‐A was successfully overexpressed as insoluble IBs in the bacterial cells (Figure 1a). After solubilization of the IBs with 6 M GdnHCl and reduction with DTT, all Cys residues reacted with TAPS‐Sulfonate to form reversible alkyl disulfides, yielding TAPS‐VEGF‐A proteins. All thiol groups were eliminated from the reaction mixtures because of the high reactivity of the methanethiosulfonate group present in TAPS‐Sulfonate with thiol groups.^32^ The inherent high cationicity of VEGF‐A was further enhanced by *the S*‐cationization of all cysteine residues located within the molecule. A more cationic polymer, PEI, was added as a carrier at the dialysis step on removing GdnHCl to eliminate anionic contaminants from the inclusion bodies.^16^ PEI formed an electrostatic complex with the bacterial anionic contaminants, causing their aggregation; however, TAPS‐VEGF‐A remained water‐soluble. Although the solubilized TAPS‐VEGF‐A contained proteolytically degraded products, complete *S*‐cationization of all the cysteine residues was confirmed by their slow migration driven by their net cationic charge using SDS‐PAGE under non‐reducing conditions (Figure 1a,b). The recovery of TAPS‐VEGF‐A proteins reached approximately 80 mg from 0.8 L bacterial cultures. Our previous studies have reported many examples of solubilization techniques related to IBs using TAPS‐Sulfonate. Proteolytic degradation was confirmed by bacterial expression; however, the amounts of the proteolytically degraded products were usually low.^16^, ^20^, ^21^, ^33^, ^34^, ^35^

In contrast to these previous studies, our study had two limitations. First, the proteolytic degradation level of VEGF‐A in our study was high. The second was that the soluble fraction of TAPS‐VEGF‐A after the dialysis step, which showed a relatively higher absorption at 260 nm, suggested a low level of nucleic acid contamination. Cationic heparin‐binding sites in VEGF‐A can interact with anionic nucleic acids.^36^ Direct refolding of TAPS‐VEGF‐A at the purity found in our study showed a low level (<1%) of refolding yield (Figure S2); thus, we next attempted to purify intact full‐length VEGF‐A.

### 3.2 Removal of proteolytic degradants and contaminants by pre‐folding purification increases refolding yield

Water‐soluble TAPS‐VEGF‐A and its associated degradation products with low levels of nucleic acid contaminants underwent pre‐folding purification via reversed‐phase HPLC to isolate the full‐length protein that exhibited high purity (Figure 1b). Although chromatographic separation did not attain a resolution adequate for distinguishing between the degraded and full‐length variants, full‐length TAPS‐VEGF‐A of a higher purity was isolated (Figure 1c). This chromatographic profile demonstrates considerably high reproducibility, yielding an approximate recovery rate of 65%.

Given the capacity of the HPLC column (2 mg/injection), multiple injections were performed to achieve the required recovery. Scaling up the size of HPLC columns for large‐scale protein production is a reasonable option. The refolding process of IBs has now become one of the modern developments in the biopharmaceutical industry.^14^ Therefore, pre‐purification using industrial‐scale reversed‐phase HPLC can be considered as an alternative method for downstream process designs. A variety of approaches are available for reverse‐phase HPLC‐based recombinant protein purification. For instance, using an optimized slow gradient^37^ or isocratic elution^38^ has resulted in highly effective purification procedures.

TAPS‐VEGF‐A was effectively refolded into biologically active homodimeric conformations by a rapid and direct dilution process using a redox refolding buffer composed of GSH/GSSG =4:1. Cationic alkyl disulfide moieties conjugated to TAPS‐VEGF‐A are eliminated under these redox buffer conditions, initiating oxidative folding toward more thermodynamically stable states.^16^, ^19^, ^20^, ^21^ Cationic alkyl thiol groups are reduced from TAPS‐VEGF‐A work as part of the redox. To screen for a buffer that can be used for optimal folding, evaluations were performed using simple GdnHCl‐ and urea‐based recipes for protein folding.^15^, ^16^ VEGF‐A showed superior yield of folding under urea‐based folding conditions. TAPS‐VEGF‐A was refolded under optimized conditions and purified using cation‐exchange chromatography. The refolded VEGF‐A tended to form homodimeric pairings with full‐length and degraded products. Therefore, a final purification step for refolded VEGF‐A using a high‐resolution Resource‐S cation‐exchange column was required to isolate full‐length homodimeric proteins (Figure 1d). The recovery rate of the full‐length and homodimeric VEGF‐A proteins from TAPS‐VEGF‐A was 21% (Figure 1d,e). The inhibitory effect of contaminants on the refolding of VEGF‐A has been demonstrated, in part, in reconstitution experiments. The refolding yield of HPLC‐purified TAPS‐VEGF‐A (fractions 34–38 min) was reduced to 55% when mixed with prominent contaminant peaks (fractions 6–8 min) (Figure S3). Liquid chromatography with tandem mass spectrometry and ultraviolet spectral analysis revealed that these impurity peaks included various low‐molecular‐mass polar molecules such as EDTA and nucleic acids.

### 3.3 Alternative methods for recombinant VEGF‐A production

**FIGURE 2:**
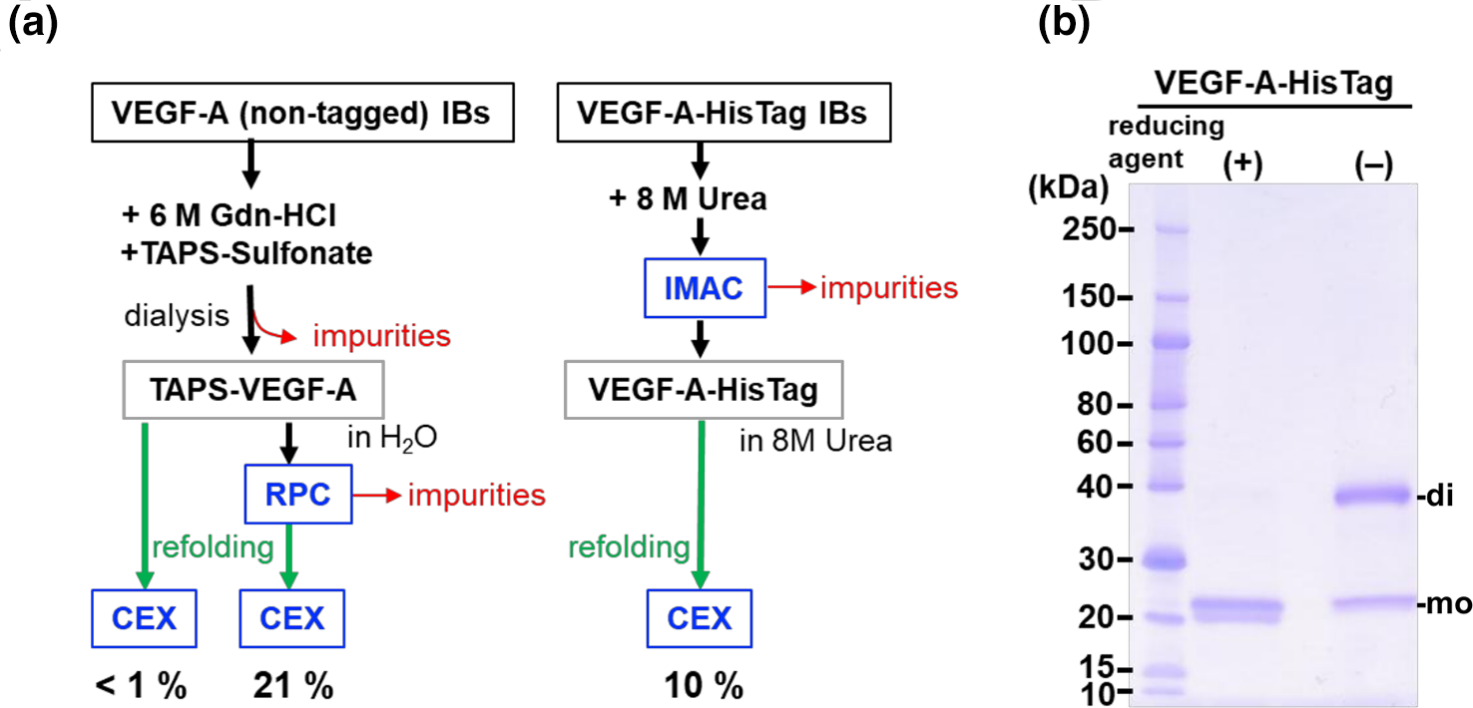
(a) Schematic representation of pre‐folding purification of non‐tagged and HisTagged VEGF‐A from IBs followed by refolding in redox buffer. The optimized procedure for TAPS‐VEGF‐A refolding requires pre‐folding purification by reversed‐phase HPLC(RPC). The refolding recovery yield for each procedure was determined by cation‐exchange chromatography (CEX) purification. (b) SDS‐PAGE analysis of VEGF‐A HisTag refolded after purification using IMAC under reducing or nonreducing conditions.

Three alternative methods were investigated in the optimization of recombinant VEGF‐A protein production methodology. The first approach involves the widely used affinity purification process of the target protein from IBs dissolved in a denaturant (Figure 2a). After pre‐folding purification procedure of the VEGF‐A‐HisTag using IMAC was performed, the purified proteins were refolded in a redox buffer. The refolded VEGF‐A–HisTag showed minimal proteolytic degradation (Figure 2b). The purified samples showed intermolecular disulfide crosslinked homodimeric conformations, albeit in the presence of contaminating misfolded monomeric products (Figure 2b). The yield of refolded purified VEGF‐A‐HisTag was approximately 10% (Figure 2a). This result underscores the effectiveness of IMAC purification in removing proteolytically degraded products, albeit with a slightly reduced redox refolding yield. Specific protease cleavage conditions would require further optimization to remove the HisTag domain from the protein.

**FIGURE 3:**
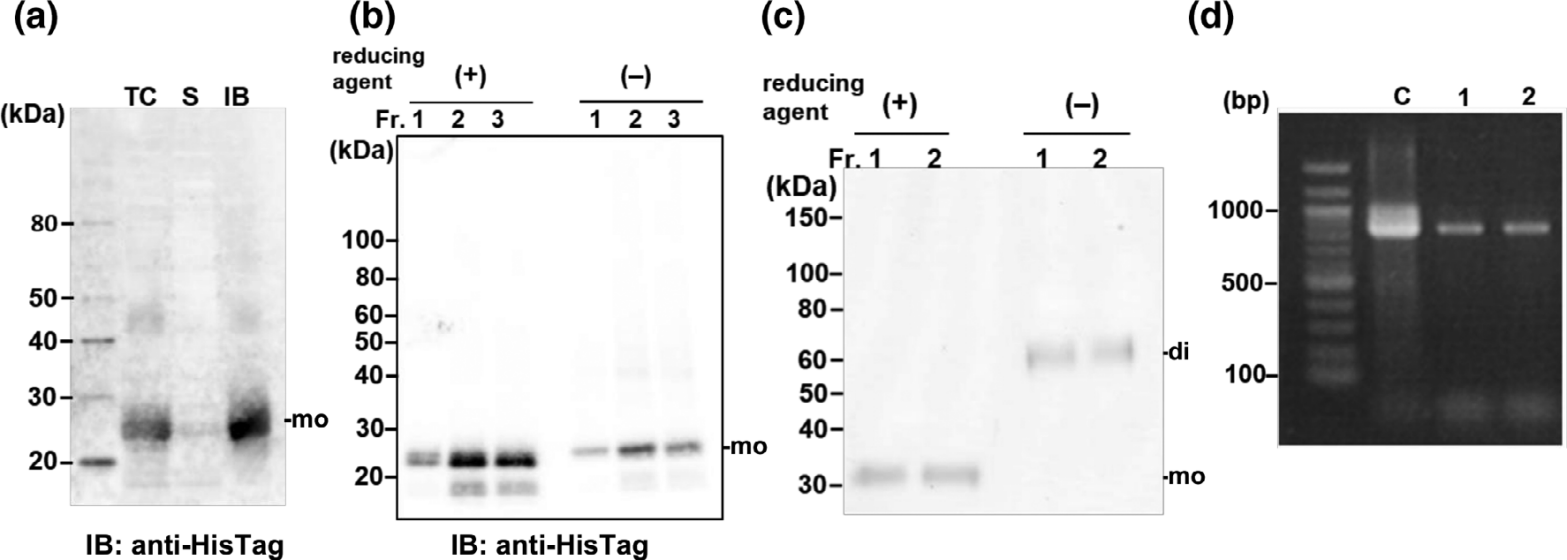
(a) Western blot analysis of VEGF‐A HisTag expressed in *E. coli* Origami2(DE3) using anti‐HisTag antibodies. (b) IMAC purification of VEGF‐A‐HisTag from soluble fractions was analyzed under reducing or non‐reducing conditions. (c) SDS‐PAGE analysis of purified VEGF‐A‐HisTag in culture media expressed in Expi293 cells using a heparin affinity column. (d) PCR amplification of VEGF‐A‐HisTag to show contaminating DNAs. Lane C, plasmid DNA control, lane 1,2, purified VEGF‐A‐HisTag using a heparin column from fractions 1 and 2 in (c).

The second approach involved in vivo folding experiments using Origami2(DE3) cells as a host. VEGF‐A HisTag protein showed partial solubilization in Origami2(DE3) cells during cultivation at 25°C for 16 h (Figure 3a). However, most of the VEGF‐A HisTag protein purified from cell lysates was in an inactive monomeric form (Figure 3b). The intricate nature of these disulfide bonds may prove to be challenging in the cytosolic environment of Origami2(DE3) cells to obtain VEGF because the VEGF protein requires the formation of three intramolecular and two intermolecular disulfide bonds to achieve biologically active conformations.^25^, ^26^

The third approach involved the expression of VEGF‐A secreted in Expi293 cells. After transient transfection of an expression plasmid DNA, the recombinant VEGF‐A HisTag protein was successfully expressed in culture media in N‐glycosylated and biologically active homodimeric forms. However, the VEGF‐A HisTag protein failed to be purified using the IMAC column because of its insufficient adsorption on the IMAC column. This is unclear and may be due to structural hindrance or bound nucleic acids covering the carboxyl‐terminal fused HisTag in VEGF‐A. It has been reported that VEGF‐A in body fluid can be purified by using heparin‐binding affinity.^39^ Thus, the VEGF‐A‐HisTag was purified based on its heparin‐binding properties (Figure 3c). Although VEGF‐A‐HisTag showed sufficient purity on SDS‐PAGE, the purified protein was contaminated with nucleic acids that showed high absorbance at 260 nm. These nucleic acids represent the plasmid DNA used for transient transfection (Figure 3d). The tight interaction between the DNA and cationic VEGF‐A HisTag (pI = 9.25) could not be dissociated either by washing with salts or DNase digestion. This tight interaction between plasmid DNA was not observed in the transient expression of EPO (pI = 8.75) using the same procedures (Figure S4), indicating that the highly cationic VEGF‐A has the property to interact unusually tightly with DNAs. Tight interactions between recombinant proteins and DNAs can interfere with their biological activities. Consequently, VEGF‐A HisTag contaminated with DNAs did not show any biological activities on HUVECs (Figure S5).

Considering all the above results, refolding from TAPS‐VEGF‐A after pre‐folding purification by reversed‐phase HPLC is the best, reaching 21% of the purification yield. Pre‐folding purification is crucial for highly cationic and complex disulfide pairing. Anionic nucleic acid contaminants interfere with protein folding and chromatographic purification of VEGF‐A.

### 3.4 Biological activities of purified VEGF‐A

**FIGURE 4:**
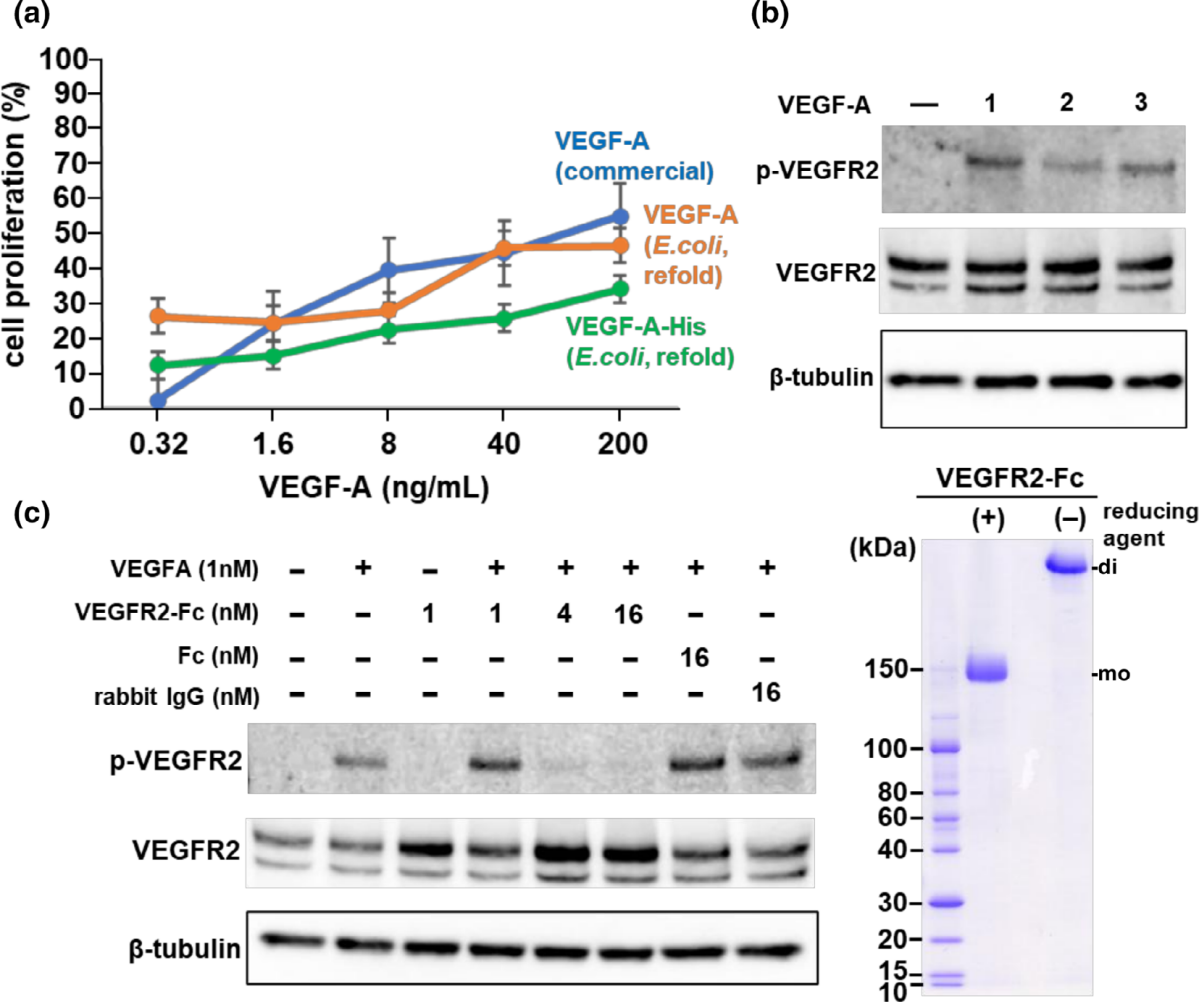
Biological activity of VEGF‐As. (a) Induction of proliferation of HUVECs by VEGF‐A was evaluated after four days of cultivation. (b) Induction of VEGFR2 phosphorylation of untagged VEGF‐A (lane 1), VEGF‐A‐HisTag (lane 2), or commercial VEGF‐A control (lane 3) was confirmed by western blotting. (c) Inhibition of VEGF‐A‐induced VEGFR2 phosphorylation by the extracellular domain of VEGFR2 Fc proteins. SDS‐PAGE analysis of recombinant VEGFR2‐Fc expressed in Expi293 cells and purified using IMAC. β‐tubulin served as a positive control.

Biological activity of the purified VEGF‐A were confirmed using HUVECs. Induction of cell proliferation of HUVECS by the purified VEGF‐A was almost comparable with other VEGFs (Figure 4a). The level of induction of VEGFR2 phosphorylation by VEGF‐A stimulation was similar to that of refolded VEGF‐A, including commercially available products (Figure 4b). This VEGF‐A–induced phosphorylation of VEGFR2 was successfully blocked by adding extracellular domains of VEGFR2 Fc proteins (Figure 4c). These results indicate that the refolded and purified VEGF‐A proteins retained their biological activities.

## 4 CONCLUSION

**FIGURE 5:**
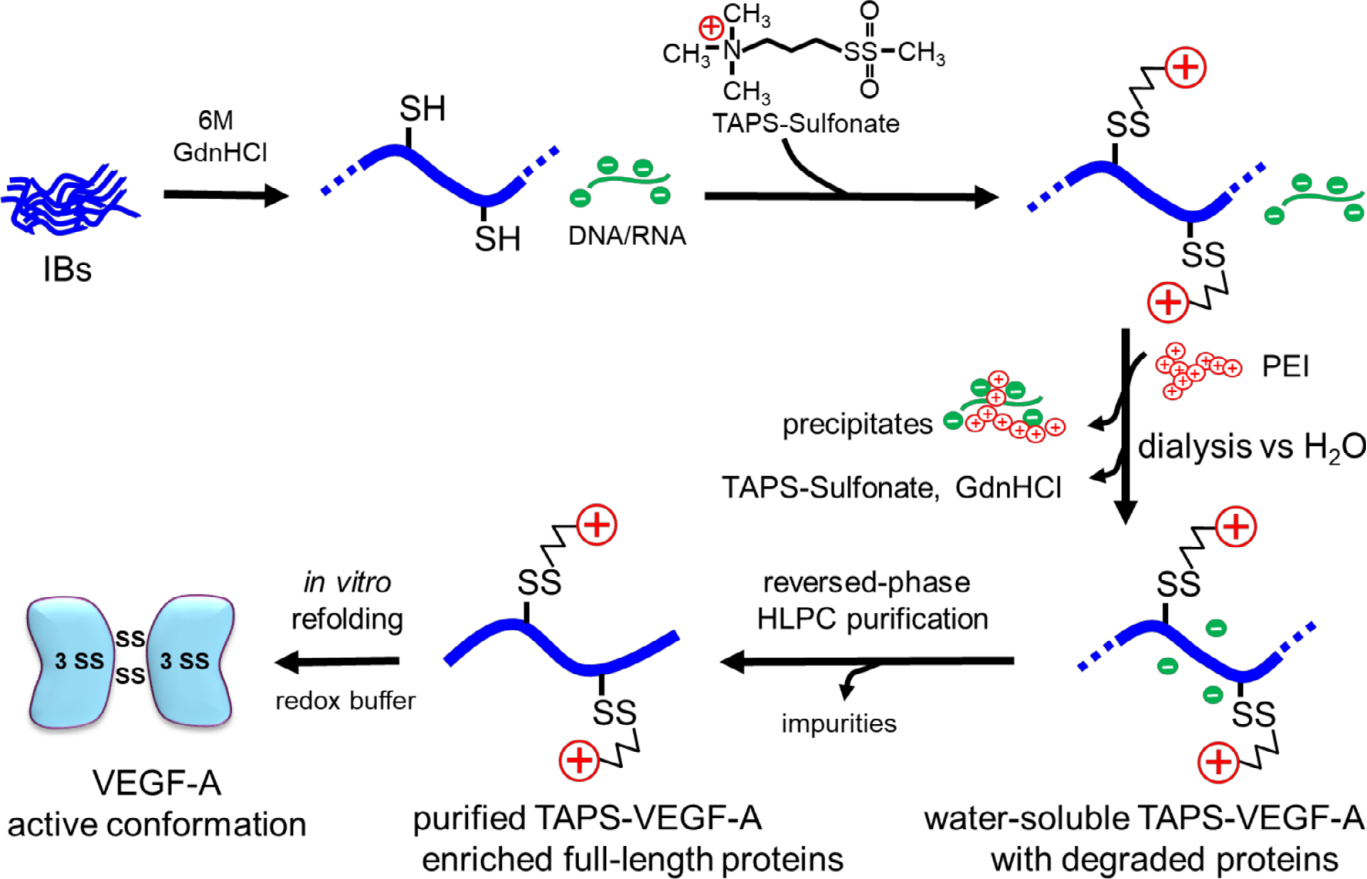
Purification procedure of pre‐folded TAPS‐VEGF‐A by reversed‐phase HPLC to remove proteolytically degraded proteins and tightly bound bacterial‐derived contaminants. Please note that water‐soluble and reversible *S*‐cationization techniques enabled refolding of the protein to form active VEGF‐A.

In this study, we have presented a robust and reliable protocol for refolding and purification of disulfide‐bonded and non‐tagged proteins from IBs (Figure 5). This method is particularly advantageous for proteins susceptible to proteolytic degradation during expression in host cells and for removal of tightly bound bacteria‐derived impurities. This approach utilizes solubilization techniques involving reversible *S*‐cationization, which enhances the hydrophilicity of target proteins. Our previous study showed that a refolding inhibitor contaminated with reversibly *S*‐cationized protein can be removed by adding PEI as a carrier during the dialysis step.^16^ However, the TAPS‐VEGF protein failed to remove an IBs‐derived refolding inhibitor (Figure S2). This critical issue was overcome by pre‐folding purification using reversed‐phase HPLC, resulting in improved refolding yields and effective removal of proteolytically degraded byproducts and impurities in the IBs. While optimal refolding conditions may vary depending on each protein,^40^, ^41^, ^42^ in our study, the reversibly *S*‐cationized proteins could be directly refolded by using a redox refolding buffer. This method is suitable for reconstituting recombinant proteins requiring non‐tagged latent forms, avoiding leakage of metal contaminants from the IMAC column.

## AUTHOR CONTRIBUTIONS

**Shuichiro Kimura:** Investigation; writing – original draft; visualization. **Wataru Yamamoto:** Investigation; visualization. **Ai Miyamoto:** Investigation. **Koreyoshi Imamura:** Writing – review and editing. **Junichiro Futami:** Conceptualization; project administration; funding acquisition; writing – review and editing.

## FUNDING INFORMATION

This work is partially supported by JSPS KAKENHI Grant Number 22H01881 (JF).

## CONFLICT OF INTEREST STATEMENT

The authors declare no conflict of interest.

## Supporting information


**Data S1.** Supplementary Information.

## Data Availability

It is available upon reasonable request.
