# Supplementary material for: Pre‐folding purification procedures for inclusion body‐derived non‐tagged cationic recombinant proteins with multiple disulfide bonds for efficient refolding
Source: Biotechnol Prog. 2025 Jan 25;41(3):e3532. doi: 10.1002/btpr.3532 (PMC12171399; doi:10.1002/btpr.3532)
Supplement: Supplementary file 1 — Data S1. Supplementary Information. [file BTPR-41-e3532-s001.pdf]

Supplementary Material

Pre-folding purification procedures for inclusion body derived non-tagged cationic recombinant proteins with multiple disulfide bonds for efficient refolding

Shuichiro Kimura[1], Wataru Yamamoto[2], Ai Miyamoto[2],

Koreyoshi Imamura[1], Junichiro Futami[2,*]

*** Correspondence: Junichiro Futami: futamij@okayama-u.ac.jp**

1. Supplemental Figures

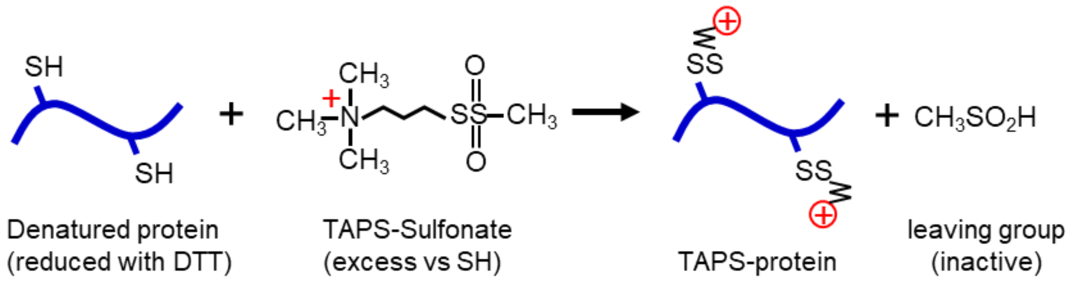

Figure S1 This is a reaction scheme for TAPS-sulfonate to Cys residues in a denatured protein by reversible S-alkyl-disulfide-cationized bonds. This reaction is conducted in a high concentration of denaturant. The leaving group of methyl sulfonic acid no longer reacts to the SH/SS exchange reaction. After dialysis out of reagents, TAPS protein shows high water solubility.

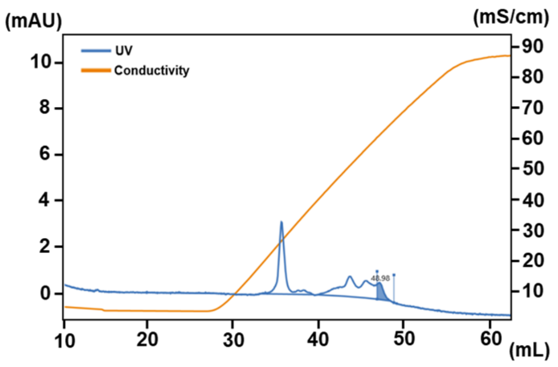

Figure S2 Cation exchange chromatography profile of VEGF-A refolded from TAPS VEGF-A without reverse-phase HPLC prefolding. The peak fraction marked in blue indicate refolded VEGF-A.

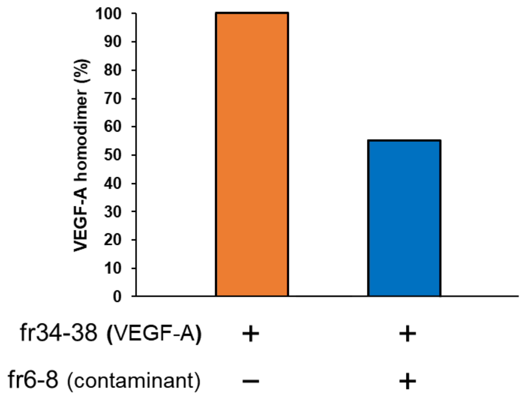

Figure S3 Effects of prominent contaminant peaks (Figure 1B, fractions collected at 34-38 min) on the refolded protein yield of HPLC-purified TAPS-VEGF-A (Figure 1B, fractions collected at 34-38 min).

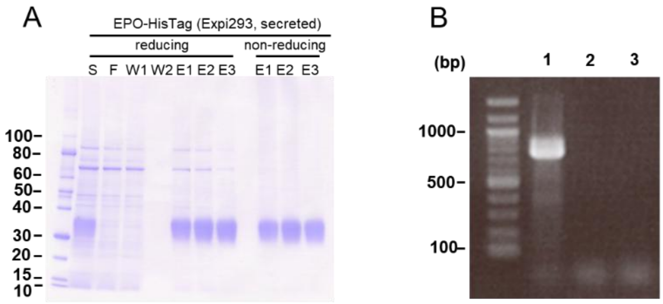

Figure S4 (A) SDS-PAGE analysis of expression and purification of EPO-HisTag expressed in Expi293 cells. Secreted EPO-HisTag in culture media (S), not observed in flow throw fraction (F) and wash fractions (W1,2), was successfully eluted (E1-E3) from the IMAC column. MW marker was used in the same as Figure 1. (B) PCR analysis of contaminant DNAs by amplification of EPO-HisTag. Lane 1, plasmid DNA control, lane 2-3, purified EPO-HisTag purified using IMAC.

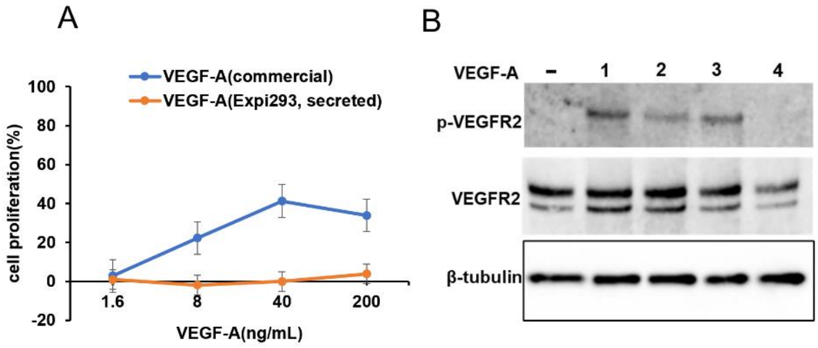

Figure S5 Analysis of biological activity of DNA-contaminated VEGF-A transiently expressed in Expi293 cells. (A) Tightly bound DNAs to VEGF-A failed to induce HUVEC proliferation after four days of cultivation. (B) DNA-contaminated VEGF-A (lane 4) failed to induce phosphorylation of VEGF-R2 compared to untagged VEGF-A (lane 1), VEGF-A-HisTag (lane 2), or commercial VEGF-A control (lane 3). Please note that this immunoblot analysis was performed on the same PVDF membrane as shown in Figure 4B.
